# The GLP-1 receptor agonists exenatide and liraglutide activate Glucose transport by an AMPK-dependent mechanism

**DOI:** 10.1186/s12967-016-0985-7

**Published:** 2016-07-30

**Authors:** Francesco Andreozzi, Gregory Alexander Raciti, Cecilia Nigro, Gaia Chiara Mannino, Teresa Procopio, Alberto M. Davalli, Francesco Beguinot, Giorgio Sesti, Claudia Miele, Franco Folli

**Affiliations:** 1 Department of Medical and Surgical Sciences, University of Catanzaro “Magna-Graecia”, Catanzaro, Italy; 2 Division of Diabetes, Department of Medicine, University of Texas Health Science Center, San Antonio, TX USA; 3 Institute of Experimental Endocrinology and Oncology “G. Salvatore”, National Council of Research, Naples, Italy; 4 Department of Translational Medical Sciences, University of Naples “Federico II”, Naples, Italy; 5 Department of Medicine Endocrinology Unit, Ospedale San Raffaele, Milan, Italy; 6 Department of Internal Medicine, University of Campinas, Campinas, SP Brazil

**Keywords:** Exenatide, Liraglutide, Glucose uptake, AMPK, Skeletal muscle cells, Insulin signaling

## Abstract

**Aims/hypothesis:**

Potentiation of glucose-induced insulin secretion is the main mechanism of exenatide (EXE) antidiabetic action, however, increased glucose utilization by peripheral tissues has been also reported. We here studied the effect of EXE on glucose uptake by skeletal muscle cells.

**Methods:**

2-deoxy-glucose (2DG) uptake and intracellular signal pathways were measured in rat L6 skeletal muscle myotubes exposed to 100 nmol/l EXE for up to 48 h. Mechanisms of EXE action were explored by inhibiting AMPK activity with compound C (CC, 40 μmol/l) or siRNAs (2 μmol/l).

**Results:**

Time course experiments show that EXE increases glucose uptake up to 48 h achieving its maximal effect, similar to that induced by insulin, after 20 min (2- vs 2.5-fold-increase, respectively). Differently from insulin, EXE does not stimulate: (i) IR β-subunit- and IRS1 tyrosine phosphorylation and binding to p85 regulatory subunit of PI-3kinase; (ii) AKT activation; and (iii) ERK1/2 and JNK1/2 phosphorylation. Conversely, EXE increases phosphorylation of α-subunit of AMPK at Thr172 by 2.5-fold (p < 0.01). Co-incubation of EXE and insulin does not induce additive effects on 2DG-uptake. Inhibition of AMPK with CC, and reduction of AMPK protein expression by siRNA, completely abolish EXE-induced 2DG-uptake. Liraglutide, another GLP-1 receptor agonist, also stimulates AMPK phosphorylation and 2DG-uptake. Moreover, EXE stimulates 2DG-uptake also by L6 myotubes rendered insulin-resistant with methylglyoxal. Finally, EXE also induces glucose transporter Glut-4 translocation to the plasma membrane.

**Conclusions/interpretation:**

In L6 myotubes, EXE and liraglutide increase glucose uptake in an insulin-independent manner by activating AMPK.

**Electronic supplementary material:**

The online version of this article (doi:10.1186/s12967-016-0985-7) contains supplementary material, which is available to authorized users.

## Background

Glucagon-like peptide-1 (GLP-1) is a gut hormone secreted by intestinal L cells into the bloodstream in response to nutrient ingestion and, together with the glucose-dependent insulinotropic peptide (GIP), belongs to the incretins family [1, 2]. Exenatide (EXE) is the synthetic analog of exendin-4, a GLP-1 homologous originally purified from the salivary glands of the lizard *Heloderma suspectum*, and it is currently employed in the treatment of type 2 diabetes (T2D) [3, 4]. In contrast to GLP-1, which is rapidly cleaved by the dipeptidyl peptidase IV (DPP-IV) into its inactive form, EXE is resistant to the proteolytic effect of DPP-IV, has comparable effects on insulin secretion and exerts a more potent blood glucose lowering effect [3–7].

GLP-1 and EXE stimulate glucose-dependent insulin secretion and decrease glucagon release after binding to the GLP-1 Receptor (GLP-1R) present on pancreatic endocrine β- and α-cells [8, 9]. The GLP-1R is coupled to a G protein that, once activated, increases intracellular cyclic AMP (cAMP) and induces activation of protein kinase A (PKA), extracellular signal-regulated kinase (ERK)1/2 and phosphoinositol 3 kinase (PI3K)/protein kinase B (PKB) [10–12]. It has been previously shown that GLP-1 exerts also an insulin-sensitizing effect by stimulating the uptake of glucose via the activation of PI3K, AKT and p70s6 k, whereas EXE seems to share only in part this capability in human myocytes [13–16].

The heterotrimeric protein AMPK, consisting of a catalytic α and regulatory β and γ subunits, is ubiquitously expressed and it is regulated by a variety of physiological stimuli and drugs such as metformin and thiazolidinediones and plays a pivotal role in the regulation of mitochondrial biogenesis, fatty acid synthesis and glucose uptake [17]. Experiments performed in human skeletal muscle cells have shown that AMPK activation, through phosphorylation at threonine 172 (T172) of the α subunit and subsequent phosphorylation of TBC1D4 (AS160) and TBC1D1, induces the translocation of the glucose transporter Glut-4 to the plasma membrane [18–22].

To learn more about the influence of GLP-1R agonists administration on peripheral glucose utilization, we here studied the effects of EXE and liraglutide (lira) on the molecular mechanisms leading to glucose uptake in rat L6 myotubes.

## Methods

### Reagents

Media, sera and antibiotics for cell culture were from Lonza (Walkersville, MD, USA). Protein electrophoresis and western blot reagents were from Bio-Rad (Richmond, VA, USA) and electrochemiluminescence reagents from Pierce (Rockford, IL, USA). Insulin was from Eli Lilly (Florence, Italy). The antibodies used were: anti-GLP-1 receptor (kind gift of Drs. Wanda Dolci and Bernard Thorens [10, 23], anti-insulin receptor (IR), anti-p-tyrosine (Biosource, Camarillo, CA), anti-IRS-1 (Millipore, Billerica, MA, USA), anti-p85 subunit of PI3-kinase, anti-Akt, anti-p-Akt S473, anti-p-AMPK T172, anti-AMPK, anti-p-ACC S78/80, anti-ACC, anti-p-JNK T183/Y185, anti-JNK, anti-p-AS160T642, anti-AS160 (Cell Signaling Technology, Beverly, MA, USA), anti-ERK1/2, anti-p-ERK1/2 (Santa Cruz, CA, USA), anti-GLUT4 antibodies [24], and anti-αTubulin (Sigma, St Louis, MO, USA). 5-amino-4-imidazole carboxamide riboside (AICAR), Compound C, and LY294002 were from Calbiochem (La Jolla, CA). SiRNAs were from Riboxx (Radebeul, Germany). Methylglyoxal (MGO) was from SIGMA-Aldrich (St Louis, MO, USA). Exenatide and liraglutide were kind gifts from Amylin and Novo Nordisk, respectively.

### Cell culture, treatment and transfection of siRNAs

Rat L6 skeletal muscle myoblasts were grown in complete high glucose DMEM (with l-glutamine) supplemented with 10 % (vol/vol) inactivated fetal calf serum and 1 % (vol/vol) antibiotics-antimycotics and maintained in a humidified atmosphere of 5 % CO2 in air at 37 °C. L6 myotubes were allowed to differentiate as described previously [25, 26]. Myotubes were treated with EXE 100 nmol/l for 20 min and 2, 4, 24 and 48 h, or with insulin 100 nmol/l for 30 min. Where indicated, cells were treated with AICAR 2 mmol/l for 2 h, or pre-treated with CC 40 μmol/l for 30 min, with LY 25 μmol/l for 30 min, methylglioxal 2.5 mmol/l for 30 min. L6 myotubes were also transfected with siAMPK-1, -2 and -3 targeting both AMPK α 1 and 2, using Oligofectamine Reagent from Life Technologies (Carlsbad, CA) according to the manufacturer’s instructions as previously described [27]. SiNR was used as negative control.

### Western blot analysis

Baboons tissues and human skeletal muscle proteins were extracted as previously described [28, 29]. α-TC1 and β-TC3 cell lines were a kind gift of prof. Douglas Hanahan [30]. L6 myotubes protein were obtained by lysing cells in buffer containing 50 mmol/L HEPES (pH 7.5), 150 mmol/L NaCl, 10 mmol/L EDTA, 1 % Triton X-100, 10 mmol/L Na_4_P_2_O_7_, 100 mmol/L NaF, and 2 mmol/L sodium orthovanadate supplemented with protease inhibitors cocktail. Protein concentration was determined with the Bradford assay (DC Protein Assay; Bio-Rad, Hercules, CA) according to the manufacturer’s instructions. For the immunoprecipitation protocol, equal amounts of proteins (300 µg) were incubated at 4 °C overnight with anti-IRS1 antibody; immune complexes were collected by incubation with protein A-sepharose for 3 h at 4 °C and resuspended in Laemmli buffer. Equal amounts of proteins resolved by SDS-PAGE were electrophoretically transferred to nitrocellulose membrane (Amersham Biosciences, Piscataway, NJ). The membranes were incubated with primary antibodies followed by incubation with peroxidase-conjugated secondary antibodies. Proteins were detected by using enhanced chemiluminescence (Amersham Biosciences, Piscataway, NJ), and band densities were quantified by densitometry. To normalize the blots for protein levels, after being immunoblotted with antiphosphospecific antibodies, the same blots were stripped and re-probed with appropriate primary antibodies. To assess Glut 4 translocation to the plasma membrane upon EXE treatment, plasma membrane proteins were extracted from L6 myotubes by the use of “Mem-PER Plus Membrane Protein Extraction Kit” (Thermo Fisher Scientific, Rockford, IL, USA) following the manufacturer instructions. Equal amount of protein extracts were then analyzed by western blot as previously described in this section.

### Determination of 2-deoxyglucose (2DG) uptake

2-DG uptake measurements were carried out as described previously [25, 26]. Myotubes were starved for 24 h and, subsequently, treated with hormones or chemical compounds. Cells were supplemented during the final 10 min with 0.2 mmol/L 2-DG and then solubilized. 2-DG uptake was quantified by liquid scintillation counting.

### Gene expression analysis

Total RNA was obtained from L6 myotubes, reverse transcribed using the high capacity cDNA Reverse Transcription Kit (Applied Biosystems, Foster City, CA) and analyzed by Real-Time quantitative PCR using a Power SYBR Green PCR Master Mix (Applied Biosystems, Foster City, CA) with 10 ng of cDNA and 0.3 uM of the following oligonucleotide primers: GLUT4 FW: 5′-CCACAGAAAGTGATTGAACAGAGC-3′ GLUT4 RV: 5′-AATGATGCCAATGAGAAAGGAG-3′. The rat beta-2 microglobulin (B2M) was amplified and used to normalize the results according to the Livak method, with the following oligonucleotide primers: B2M FW: 5′-ACTGAATTCACACCCACCGA-3′; B2 m rv: 5′-attacatgtctcggtcccaggt-3′.

### Statistical analysis

All results are given as mean ± SD. Statistical differences were assessed by Student’s t test and ANOVA. A p ≤ 0.05 was considered statistically significant.

### Ethics

All animal studies (non-human primate) were approved by the Texas Biomedical Research Institute Animal Care and the Use Committee as well as the University of Texas Health Science Center at San Antonio (San Antonio, TX, USA), Institutional Animal Care and Use Committee. Human studies were approved by the Institutional Review Board of University of Texas Health Science Center at San Antonio (San Antonio, TX, USA).

## Results

### Glp-1 receptor expression in different species and tissues

**Fig. 1:**
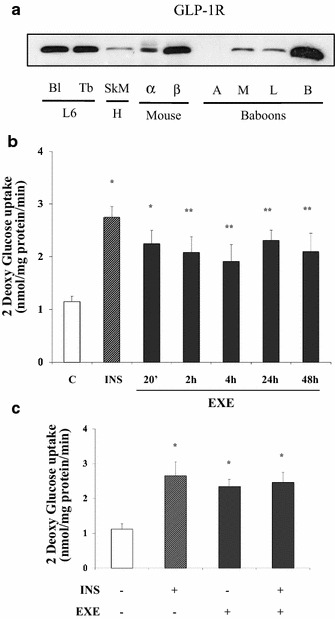
GLP-1R expression in different species and tissues: **a** Murine endocrine islet cells (αTC1 and βTC3), rat L6 myoblast (Bl) and myotube (Tb), human skeletal muscle (M) and baboon adipose (A) muscle (M) liver (L) and brain (B). Time course of EXE-induced 2DG uptake in L6 myotubes **b** Myotubes were stimulated with 100 nmol/l for 20 min, and 2, 4, 24, and 48 h and with 100 nmol/l insulin (INS) for 30 min as control. Effects of co-incubation with EXE and insulin on 2DG-glucose uptake **c** Myotubes were stimulated with either 100 nmol/l EXE, 100 nmo/l INS and both. Data are shown as mean ± SD of three independent experiments. (*p < 0.001, **p < 0.01 vs control)

EXE, as well as other GLP-1 receptor agonists, exert their function by binding to the GLP-1R. Thus, we evaluated by Western Blot analysis whether GLP-1R protein was expressed in undifferentiated (myoblast) and differentiated (myotubes) L6 rat skeletal muscle cells. Baboon adipose tissue, skeletal muscle, liver and brain, human gastrocnemius, α-TC1 and β-TC3 cell lines were used as control of expression. GLP-1R was highly expressed in myotubes with levels comparable to those observed in baboon brain (B). GLP-1R was also present in human gastrocnemius and in baboon skeletal muscle (M), adipose (A) and liver (L) baboon tissues, in α- and β-TC3 cells (Fig. 1a).

### EXE induces 2-deoxy-d-glucose (2DG) uptake in L6 myotubes

Differentiated L6 cells were exposed to 100 nmol/l EXE from 20 min (acute stimulation) up to 48 h (chronic stimulation). Exposure to 100 nmol/l insulin for 30 min served as positive control. EXE induced a 2-fold increase in 2DG uptake already after 20 min and 2DG uptake remained steadily higher than in control untreated cells up to 48 h (Fig. 1b). Noteworthy, after acute stimulation, the effect of EXE did not differ from that of insulin. We then investigated whether the effect of EXE on glucose uptake was additive to that of insulin. Co-incubation with EXE and insulin did not further increase 2DG uptake as compared to either insulin or EXE alone (Fig. 1c).

### EXE induces AMPKα activation in myotubes

**Fig. 2:**
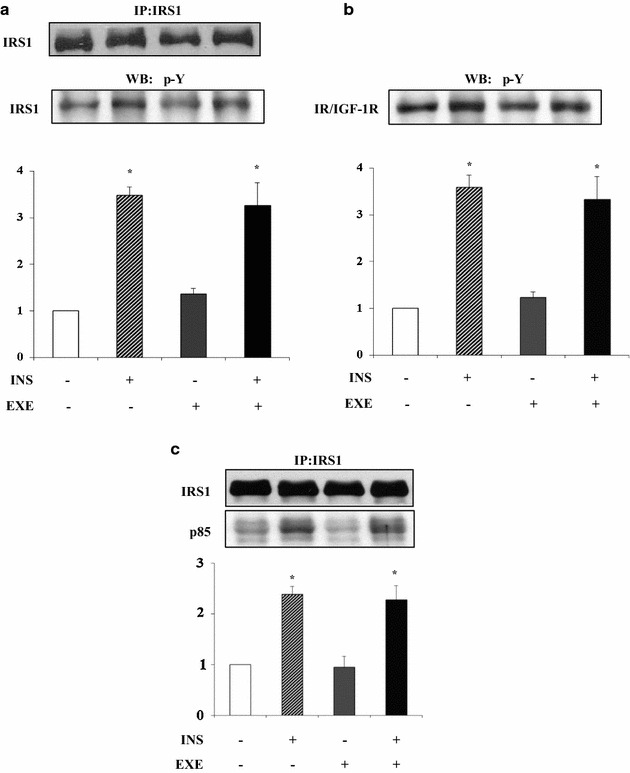
IRβ/IRS1/p85 signaling pathway in L6 myotubes. Cells were stimulated with either 100 nmol/l EXE, 100 nmol/l INS and both. The cells were then lysed and equal amounts of total proteins were immunoprecipitated with anti-IRS-1 antibody and immunoblotted with antiphosphotyrosine antibody (**a, b**) and anti-p85 subunit of PI 3-kinase (**c**). To normalize for protein levels the blots were stripped and re-probed with anti-IRS-1 antibody (A and C, top panel). Data are shown as mean ± SD of three independent experiments. (*p < 0.01 vs control)

**Fig. 3:**
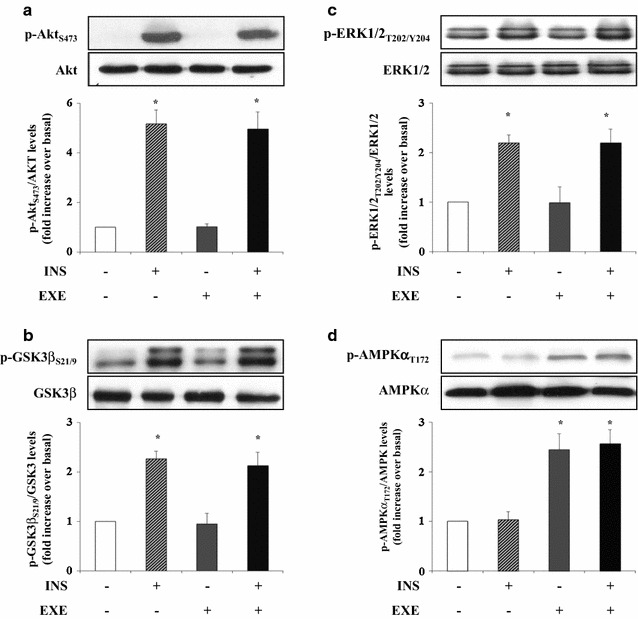
AMPKα activation in L6 myotubes. Cells were stimulated with either 100 nmol/l EXE, 100 nmol/l INS and both. After stimulation, whole lysates were immunoblotted with the phospho-specific antibodies: p-Akt S473 (**a**), p-GSK3β S21/9 (**b**), p-ERK1/2 T202/Y204 (**c**) and p-AMPK T172 (**d**). In order to normalize for protein levels, blots were stripped and re-probed with anti-AKT, anti ERK1/2, anti-GSK3β and anti-AMPK antibodies. Data are shown as mean ± SD of three independent experiments. (*p < 0.001 vs basal)

We then investigated the molecular mechanisms involved in EXE-induced glucose uptake. Differentiated L6 muscle cells were treated with either EXE for 20 min, insulin for 30 min, or both. In contrast to insulin, EXE did not induce tyrosine phosphorylation of either IR β-subunit and IRS-1 (Fig. 2a, b). Because association of the p85 regulatory subunit of PI3K with tyrosine-phosphorylated IRS-1 is essential to promote downstream PI3 kinase signaling, the effect of EXE on IRS-1 docking to p85 regulatory subunit of PI3K was examined by immunoprecipitation of IRS-1 from cell lysates followed by immunoblotting with anti-p85 antibodies. IRS-1 tyrosine phosphorylation following insulin stimulation caused increased association with the p85 regulatory subunit while this was not detected after EXE treatment (Fig. 2c). Furthermore, EXE did not activate AKT and its substrate GSK3β, as determined by assessing phosphorylation at Ser473 and Ser21/9 respectively (Fig. 3a, b). Additionally, EXE treatment did not affect phosphorylation of ERK1/2 (Fig. 3a, b, c). Moreover, EXE stimulated AMPKα phosphorylation at Thr172 by 2.5 fold as compared to untreated control cells while insulin did not (Fig. 3d). Insulin and EXE did not exhibit additive effects on AKT or AMPK activation when used in combination (Fig. 3a, d), suggesting that EXE exerts its stimulatory effect on glucose uptake by an insulin-independent mechanism and possibly via AMPK activation.

### EXE stimulates AMPKα in a time and dose dependent manner

**Fig. 4:**
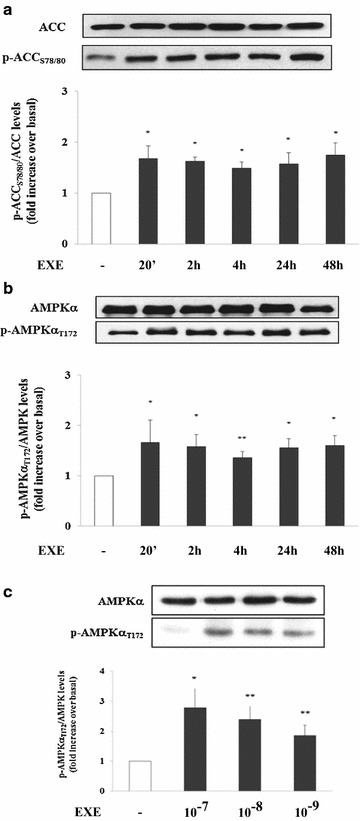
Time-course and dose–response experiments of EXE effects on AMPK and ACC phosphorylation in L6 cells. Phosphorylated and total (**a**, **b**, **c**, *upper* panels) ACC S78/80 (**a**) and AMPKa T172 (**b**) protein levels were measured in cells exposed to EXE for increasing time periods. For the dose response curve (**c**) cells were incubated for 20 min with 0, 1, 10 and 100 nmol/l EXE. Thr172 phosphorylation and total levels of AMPK were shown. Each *bar* represents the mean ± SD of three independent experiments. (*P < 0.01 and **P < 0.02 vs. control)

The time course effect on AMPKα activation and activity was examined by exposing L6 myotubes to EXE for 20 min, and 2, 4, 24, and 48 h. AMPKα phosphorylation at Thr172 and AMPK substrate ACC at Ser78/80 were determined by western blot. EXE stimulated both AMPK and ACC phosphorylation already after 20 min of exposure and these stimulatory effects were maintained up to 48 h (Fig. 4a, b). EXE significantly stimulated AMPK phosphorylation in a dose-dependent manner from 1 to 100 nmol/l (Fig. 4c). Pre-treatment of L6 myotubes for 30 min with the AMPK inhibitor CC abolished the stimulatory effect of both EXE and 2 mmol/l AICAR (a chemical activator of AMPKα) on AMPKα and ACC phosphorylation (Additional file 1: Figure S1a, b). By contrast, the stimulatory effects of EXE and AICAR on AMPKα and ACC phosphorylation, were not affected by pre-treatment with the PI3 K inhibitor LY294002 (Additional file 1: Figure S1a, b) indicating that EXE-induced AMPK activation is not mediated by the PI3K signaling pathway.

### EXE induces 2DG uptake via AMPKα activation in myotubes

To further explore the role of AMPKα activation on EXE-induced glucose uptake, 2DG uptake was measured in L6 myotubes exposed for 30 min in the presence or absence of CC (40 µmol/l) or LY294002 (25 µmol/l) and then incubated with EXE, insulin or AICAR. CC pre-treatment abolished glucose uptake stimulated by both EXE and AICAR (p < 0.001) while insulin-induced glucose uptake was only partially inhibited (Additional file 1: Figure S1c). By contrast, LY294002 abolished insulin stimulated glucose uptake while it did not impair neither EXE nor AICAR stimulated glucose uptake (p < 0.001; Additional file 1: Figure S1c). Altogether these data suggest that AMPKα activation by EXE induces glucose uptake.

**Fig. 5:**
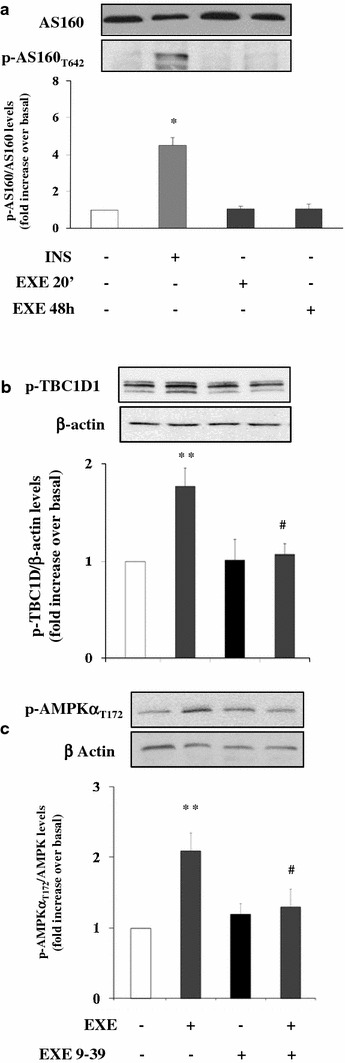
Effects of EXE (20 min or 48 h) and exendin 9–39 on AS-160, TBCD1D and AMPK in differentiated L6 cells. L6 myotubes were stimulated for 20 min with 100 nmol/l EXE, 100 nmol/l exendin 9–39 or the combination of both. Where indicated cells were stimulated with 100 nmol/l INS for 30 min and 100 nmol/l EXE for 20 min or 48 h. The blots were probed with phospho-specific AS160 T642 (**a**), TBCD1 (**b**) and AMPKα T172 (**c**). To normalize for protein levels, the blots were stripped and re-probed with β-actin antibodies. Each *bar* represents the mean ± SD of three independent experiments. (*p < 0.001 and **p < 0.01 vs. control; ^#^p < 0.02 vs. EXE)

Exposure of L6 myotubes for 30 min to CC (40 mmol/l) resulted in a marked reduction (p < 0.001) of AMPK phosphorylation in response to acute (20 min) or chronic (48 h) treatment with EXE (Additional file 1: Figure S1d). Exposure to LY294002 for 30 min did not affect AMPK phosphorylation in response to EXE (Suppl. Figure 1d). Insulin stimulated AS160 phosphorylation at Thr642, while EXE did not (Fig. 5a). Interestingly however, EXE increased by ~70 % the phosphorylation of TBC1D1, a AS160 paralog, which is more abundantly expressed in skeletal muscle and more strongly phosphorylated by AMPK, as compared to AKT, downstream insulin signaling [19–21]. This effect of EXE was abolished by its antagonist exendin 9–39 (Fig. 5b). Moreover exendin 9–39, which specifically antagonizes EXE action by binding to the GLP1 receptor, completely abolished EXE-induced AMPK phosphorylation (Fig. 5c). In addition, acute and chronic exposure to EXE did not change significantly Glut-4 mRNA levels as well as protein expression (Additional file 2: Figure 2a, b). Conversely, EXE increased of about 6-fold the translocation of Glut-4 to the plasma membrane at both 20 min and 48 h, accounting for the increased 2D-glucose uptake observed after EXE stimulation (Additional file 2: Figure 2c). In order to demonstrate equal membrane protein loading we measured the β subunit of the IR which is a useful marker of plasma membrane proteins. Both GLUT4 and IR are located in the plasma membrane, and, as showed in Additional file 2: Figure 2c, no effects were reported on IR expression after treatment with EXE at short and long time of exposure.

### Effects of AMPKα silencing on EXE-induced glucose uptake

**Fig. 6:**
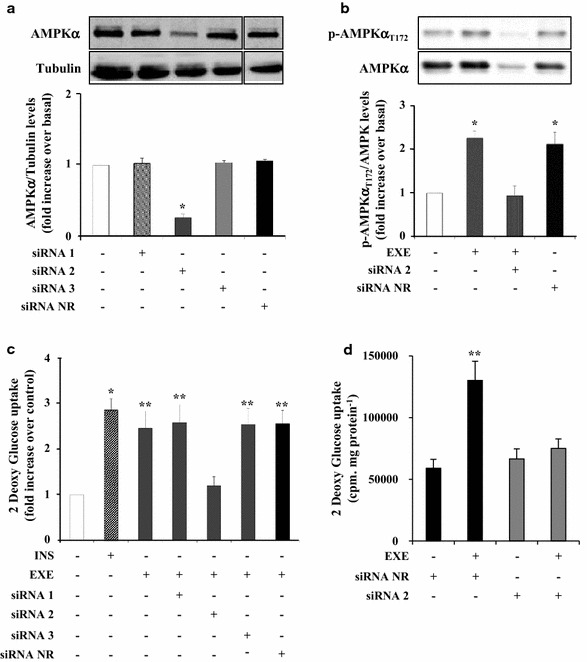
EXE induces 2DG-uptake via AMPKα activation in L6 myotubes. L6 myotubes were transfected with specific AMPK siRNAs. After transfection, the cells were stimulated with 100 nmol/l EXE or 100 nmol/l INS. Proteins were then extracted and probed with AMPKα (**a**) and phospho-specific AMPKα T172 (**b**) antibodies. To normalize the blots for protein levels, the blots were stripped and re-probed with anti-tubulin (**a**, *lower panel*) and AMPKα antibodies (**b**, *lower panel*). 2DG-uptake was measured in cells after stimulation with INS or EXE (**c**). Data are represented as fold increase over control ± SD of three independent experiments (*p < 0.001, **p < 0.01 vs basal). 2DG-uptake was also measured in cells transfected with the AMPK siRNA 2 or control SiNR, before and after stimulation with EXE. Data are shown as fold increase over control ± SD of three independent experiments (**d**) (**p < 0.01 vs basal)

The role of AMPK in EXE-mediated glucose uptake was further explored by AMPK silencing with siRNAs. AMPKα expression was reduced in L6 myotubes by using three different siRNAs targeting its α1 and the α2 catalytic subunits. Western blot analysis confirmed ~80 % down-regulation of AMPKα expression with the siRNA2 (p < 0.001; Fig. 6a) together with a marked decrease of AMPKα phosphorylation at Thr172 (p < 0.001, Fig. 6b). EXE-induced glucose uptake was abolished in L6 myotubes transfected with AMPKα siRNA2 as compared with untreated control cells or cells transfected with siRNA NR (Fig. 6c). Silencing AMPKα by using siRNA2 did not affect basal glucose uptake as compared to cells transfected with siRNA NR (Fig. 6d).

### Comparison of EXE and Liraglutide effects on glucose uptake

**Fig. 7:**
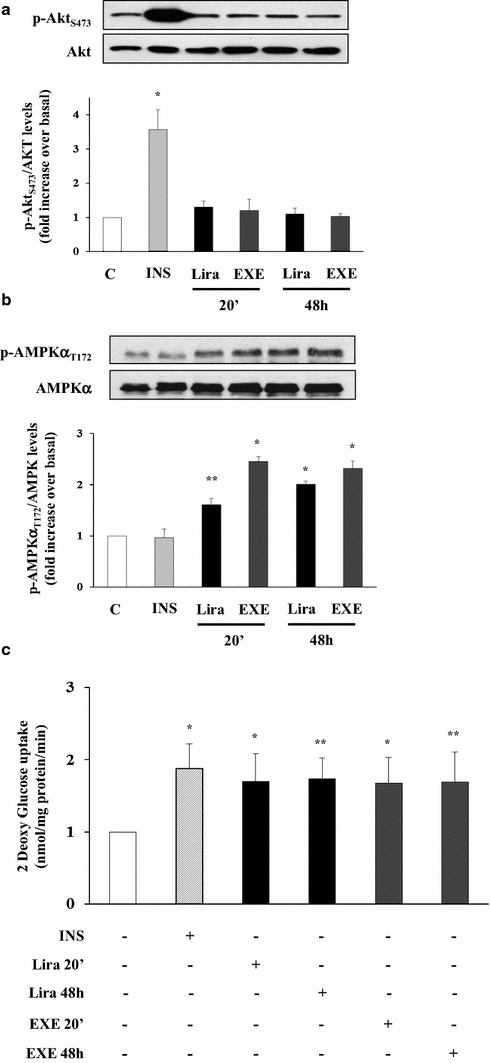
Effects of EXE and Lira on AMPK and AKT phosphorylation in differentiated L6 cells. After 20 min- and 48 h incubation with EXE and Lira, both at 100 nmol/l, levels of Thr172 (**a**) and Ser 473 (**b**) phosphorylation were measured. 2DG-uptake (**c**) was measured in L6 myotubes treated for 30 min with 100 nmol/l INS and 100 nmol/l EXE and Lira for 20 min and 48 h. Each *bar* represents the mean ± SD of three independent experiments as shown. (*p < 0.01 and **p < 0.02 vs. control)

We also explored the possibility that lira, another GLP-1R agonist, may act similarly to EXE on AKT and AMPK activation and 2DG uptake in L6 myotubes. Similarly to EXE, Lira did not stimulate AKT phosphorylation (Fig. 7a) while stimulated AMPK phosphorylation (Fig. 7b) and glucose transport (Fig. 7c). These data futher support the role of AMPK phosphorylation as a mediator of EXE and Lira effects on glucose uptake.

### Effects of EXE on MGO-induced insulin resistance in L6 myotube

**Fig. 8:**
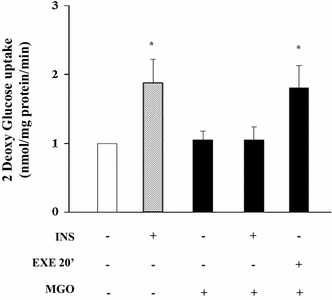
Effects of EXE on MGO-induced insulin resistance in L6 myotubes. 2DG-uptake in cultured L6 myotubes pre-incubated with 2.5 mmol/l MGO and stimulated with INS and EXE both at 100 nmol/l. Data are shown as mean ± SD of three independent experiments (*p < 0.01 vs basal and MGO)

L6 myotubes were rendered insulin resistant by using the AGE precursor MGO, an in vitro model of chronic glucose toxicity induced by protein glycation [31]. Noteworthy, EXE continued to stimulate glucose uptake in the presence of MGO, while insulin-stimulated glucose uptake in MGO treated cells was completely abolished (Fig. 8). These data further support the evidence that, in muscle cells, EXE-induced glucose transport is mediated by a molecular pathway different form that involved by insulin, and that this pathway is unaffected by MGO.

## Discussion

T2D is a chronic disease characterized by hyperglycemia, insulin resistance, defective insulin secretion and islet of Langerhans remodeling [28, 29, 32, 33]. The intestinal incretin GLP-1 also regulates glucose homeostasis in response to dietary fat and carbohydrate intake [4] and GLP-1 receptor (GLP-1R) agonists have become valuable tools in T2D treatment [34, 35].

Generally recognized mechanisms of GLP-1 and GLP-1R agonists action are potentiation of glucose-induced insulin secretion, reduction of glucagon secretion, inhibition of gastric emptying and decreased appetite [3, 4, 8, 9], however, insulin sensitizing effects have been also described. Insulin-like effects of GLP-1 have been reported in adipose tissue [36–38] and muscle cell lines [39, 40] and insulin sensitizing effects of the GLP-1R agonist EXE have been studied in animal models [41, 42] as well as in T2D subjects [43].

Previous studies in rodent [14, 44] and human cell lines [16] have shown that GLP-1 and exendin-4 can stimulate glucose uptake. In L6 myotubes, exendin-4 augments insulin-stimulated glucose uptake via a molecular pathway that requires PI3 K activation [14]. However, whether or not exendin-4 might also exert a direct stimulating effect on glucose uptake was never addressed in muscle cells. In order to address this question, we studied the effect of the exendin-4 analog EXE, given alone and in combination with insulin, on the uptake of glucose by L6 myotubes. We also explored the molecular mechanisms involved in EXE-induced glucose uptake. Our data demonstrate that EXE induces glucose uptake by utilizing a molecular pathway different from the insulin stimulated PI3K/Akt/AS160 signaling cascade. Indeed, we show that EXE has the capability to increase glucose uptake in muscle cells in the absence of insulin, and interestingly, to an extent comparable to insulin. By using CC, which is a potent chemical inhibitor of AMPK and a number of other protein kinases [45], as well as a specific siRNA-mediated knockdown designed to reduce the protein expression of the α1 and α2 subunits of AMPK, we provide direct evidence that the stimulatory effect of EXE on glucose uptake is mediated by: (i) AMPK activation; (ii) activation of the Rab GTPase-activating protein TBC1D1, a paralog of AS160, highly expressed in skeletal muscle [17, 21]; and (iii) translocation to the plasma membrane of the insulin-dependent glucose transporter Glut-4 [15, 22].

The fact that EXE stimulates glucose transport via AMPK might appear in contrast with previous data obtained in other muscle cell lines stimulated with GLP-1 in which PI3K activation has been described [5, 16]. The involvement of different pathways might be explained by the different relative abundance of PI3K and AMPK in human and mouse cell lines and the degree of cell differentiation.

AMPK is a heterotrimeric protein which mediates several cellular processes, including stimulation of glucose uptake, mitochondrial biogenesis and inhibition of fatty acid synthesis via phosphorylation of ACC [19–22]. AMPK is known to regulate adipocyte metabolism, inflammation, and vascular function and defective AMPK activation is associated with insulin resistance and T2D [46–48]. Glucose transport relies on insulin stimulated AKT phosphorylation and PI3K activation, as well as an alternative pathway involving AMPK/Cbl-CAP signaling [49]. Studies in AMPK-α1 KO mice have shown that of the two catalytic subunits of AMPK, the isoform α1 is essential for stimulation of glucose uptake by the soleus muscle [50]. The involvement of AMPK, resulting in nitric oxide synthase activation, also accounts for the improvements in endothelial dysfunction observed after EXE treatment [51, 52]. Likewise EXE-mediated beneficial effects on cardiac function are associated with AMPK activation [53].

Our findings are consistent with recent studies where EXE and Lira administration reduced fasting and postprandial blood glucose levels, and improved insulin sensitivity, in subjects with type 1 diabetes with absent endogenous insulin secretion [5, 6]. Differently from a previous report where Lira enhanced insulin sensitivity [54], we show a direct effect of EXE and Lira on glucose transport in myotubes and suggest that these GLP-1R agonists might regulate glucose homeostasis also in an insulin-independent manner through an AMPK-dependent pathway.

The lack of additive effects of EXE (and Lira) and insulin on glucose uptake can be explained by considering that the final step of their mechanism of action involves Glut-4 translocation to the plasma membrane which is already maximally stimulated by insulin. Our findings are in agreement with a previous study where Lira was reported to induce Glut-4 translocation via AMPK activation in C2C12 murine muscle cells over-expressing Glut-4, although in that study glucose transport was not evaluated [55].

It is important to note that EXE increases glucose uptake also in L6 myotubes previously treated with methylglyoxal (MGO), which is increased in hyperglycemic conditions and induces insulin resistance in these cells [31]. Altogether, our findings demonstrate that the GLP-1R agonists EXE and Lira increase glucose uptake by muscle cells in an insulin-independent manner.

## Conclusions

We demonstrate that EXE and Lira directly stimulate glucose uptake by skeletal muscle cells through AMPK and TBC1D1 phosphorylation and subsequent Glut-4 translocation to the plasma membrane. The EXE induced glucose transport is not affected by MGO-induced non-enzymatic glycosylation which, conversely, impairs insulin-stimulated glucose transport. In type 2 diabetes mellitus which is characterized by increased non-enzymatic protein glycosylation because of chronic hyperglycemia, GLP-1R agonists might also improve defective glucose transport and glycometabolic control by stimulating AMPK phosphorylation.

## Additional files

10.1186/s12967-016-0985-7 EXE induced AMPKα activation and 2DG-uptake in cultured L6 myotubes. Myotubes were pre-treated with 40 mmol/l CC or with 25 mmol/l LY for 30 min and then stimulated with 100 nmol/l EXE (for 20 min or 48 h, where indicated) or 2 mmol/l AICAR. Immunoblots of whole cell lysates and 2DG uptake were performed. The blots were probed with phospho-specific AMPKα T172 (panel A and D), and ACC S78/S80 (panel B) antibodies. To normalize for protein levels, blots were stripped and re-probed with total AMPK and ACC protein antibodies (A, B and D, lower panel). After stimulation, 2DG-uptake was measured (panel C). Data are shown as mean ± SD of three independent experiments (n = 3) (*p < 0.001, **p < 0.01 vs basal).
10.1186/s12967-016-0985-7 Effects of EXE on Glut-4 in cultured L6 myotubes. Myotubes were stimulated with 100 nmol/l EXE for 20 min or 48 h. Panel A shows qPCR of Glut-4 mRNA. In panel B is a representative western blot for Glut-4 and β-Actin (loading control). In panel C is a representative western blot for Glut-4 and β-IR (loading control) in plasma membrane (PM) extracts (Glut-4 translocation). For A and C panels, data are shown as fold increase over control ± SD of three independent experiments (*p < 0.001, vs Ctrl).

## Abbreviations

AICAR: 5-amino-4-imidazole carboxamide riboside
AMPK: AMP-activated protein kinase
cAMP: cyclic AMP
DPP-IV: dipeptidyl peptidase IV
EXE: exenatide
ERK: extracellular signal-regulated kinase
GIP: glucose-dependent insulinotropic peptide
GLP-1: glucagon-like peptide-1
GLP-1R: GLP-1 receptor
Lira: liraglutide
MAPK: mitogen-activated protein kinase
MGO: methylglyoxal
PI3 K: phosphatidylinositol 3-kinase
PKA: protein kinase A
PKB: protein kinase B
T2D: type 2 diabetes
