# Supplementary figures and images for: The GLP-1 receptor agonists exenatide and liraglutide activate Glucose transport by an AMPK-dependent mechanism
Source: J Transl Med. 2016 Jul 30;14:229. doi: 10.1186/s12967-016-0985-7 (PMC4967343; doi:10.1186/s12967-016-0985-7)

## Slide 1
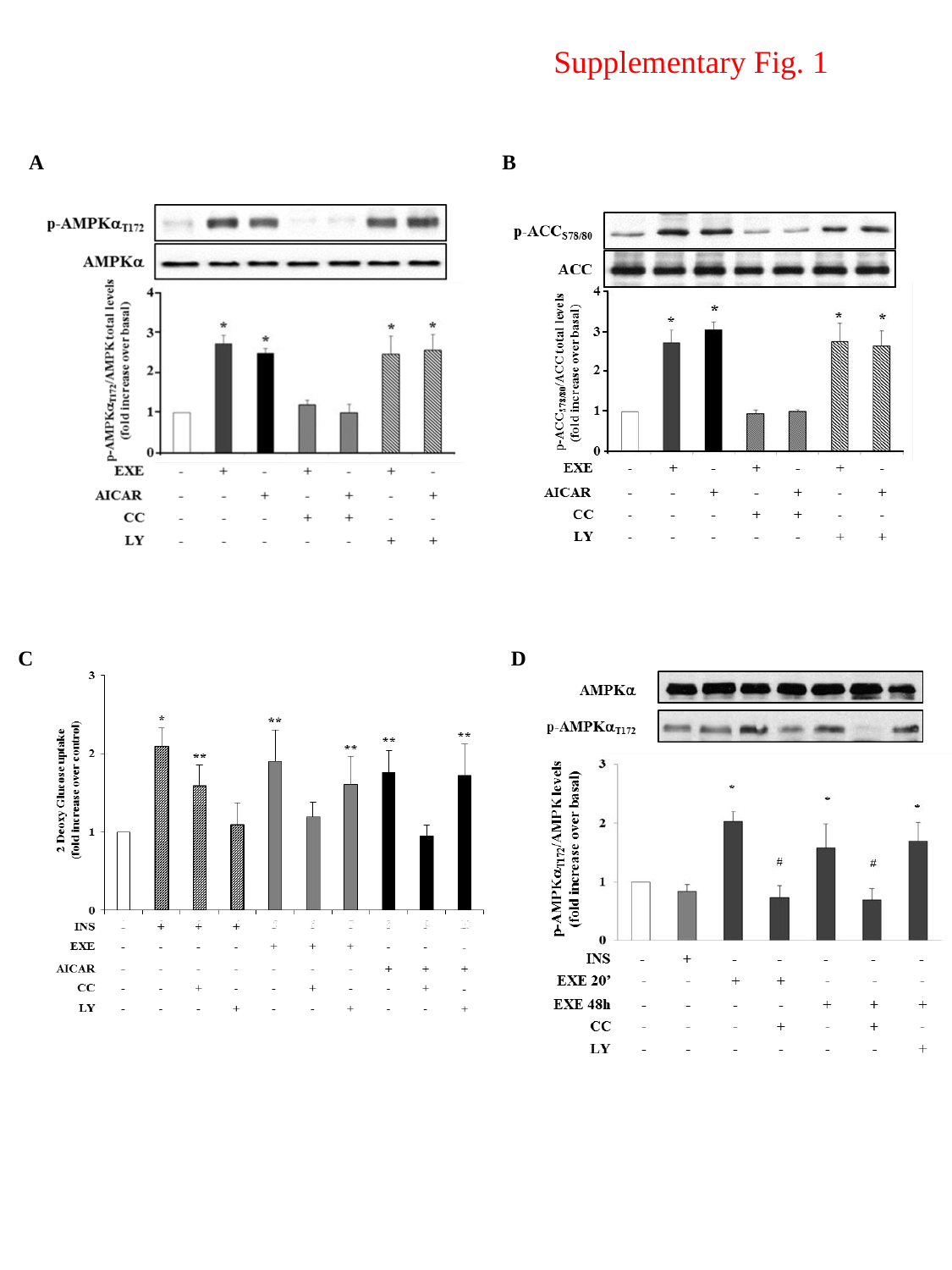

Supplementary Fig. 1
A
B
C
D

Supplement: Supplementary file 1 — 10.1186/s12967-016-0985-7 EXE induced AMPKα activation and 2DG-uptake in cultured L6 myotubes. Myotubes were pre-treated with 40 mmol/l CC or with 25 mmol/l LY for 30 min and then stimulated with 100 nmol/l EXE (for 20 min or 48 h, where indicated) or 2 mmol/l AICAR. Immunoblots of whole cell lysates and 2DG uptake were performed. The blots were probed with phospho-specific AMPKα T172 (panel A and D), and ACC S78/S80 (panel B) antibodies. To normalize for protein levels, blots were stripped and re-probed with total AMPK and ACC protein antibodies (A, B and D, lower panel). After stimulation, 2DG-uptake was measured (panel C). Data are shown as mean ± SD of three independent experiments (n = 3) (*p < 0.001, **p < 0.01 vs basal). [file 12967_2016_985_MOESM1_ESM.pptx]
