# Supplementary material for: The GLP-1 receptor agonists exenatide and liraglutide activate Glucose transport by an AMPK-dependent mechanism
Source: J Transl Med. 2016 Jul 30;14:229. doi: 10.1186/s12967-016-0985-7 (PMC4967343; doi:10.1186/s12967-016-0985-7)
Supplement: Supplementary file 2 — 10.1186/s12967-016-0985-7 Effects of EXE on Glut-4 in cultured L6 myotubes. Myotubes were stimulated with 100 nmol/l EXE for 20 min or 48 h. Panel A shows qPCR of Glut-4 mRNA. In panel B is a representative western blot for Glut-4 and β-Actin (loading control). In panel C is a representative western blot for Glut-4 and β-IR (loading control) in plasma membrane (PM) extracts (Glut-4 translocation). For A and C panels, data are shown as fold increase over control ± SD of three independent experiments (*p < 0.001, vs Ctrl). [file 12967_2016_985_MOESM2_ESM.pptx]

## Slide 1
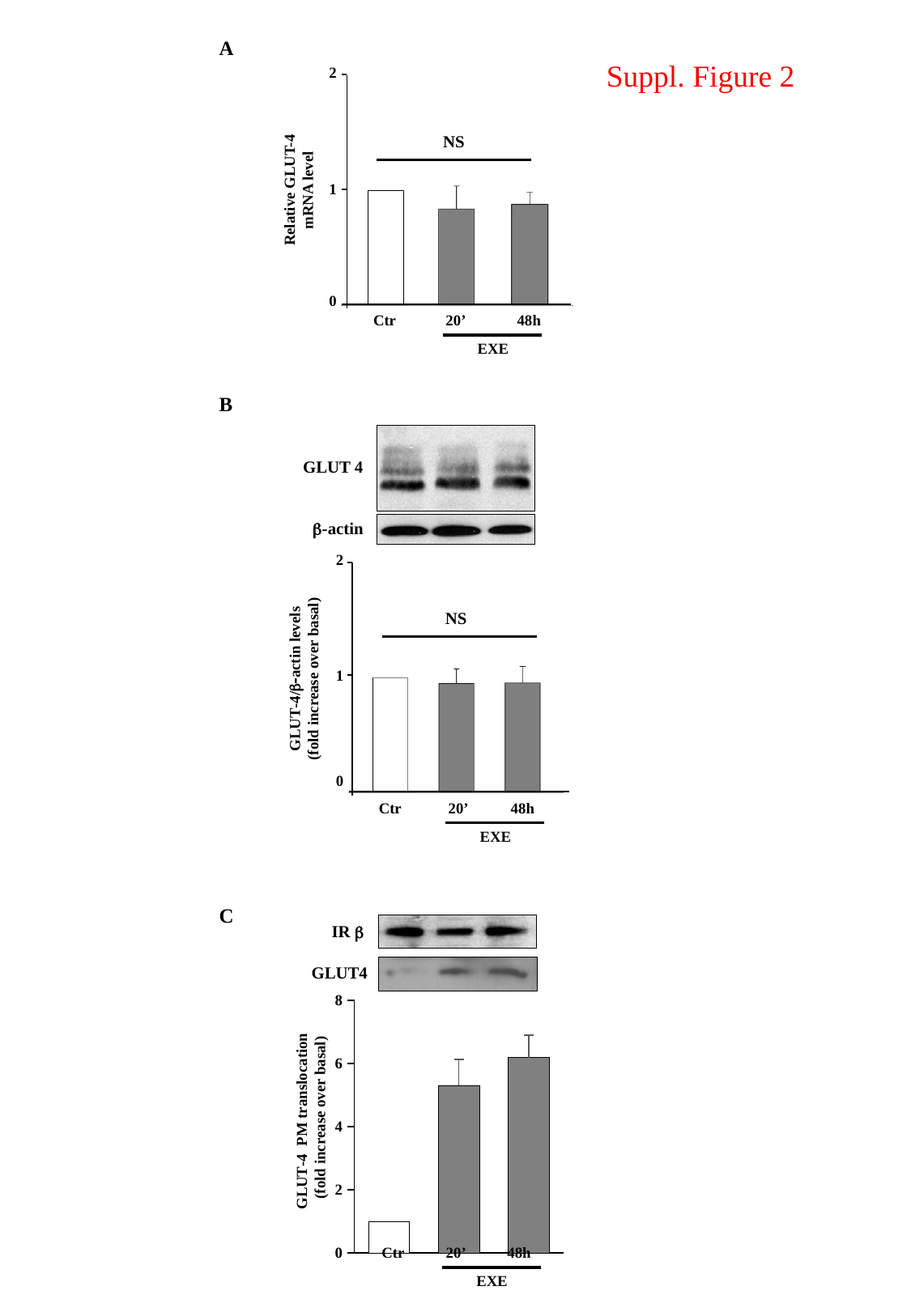

A
2
NS
Relative GLUT-4
mRNA level
1
0
Ctr
20’
48h
EXE
Suppl. Figure 2
B
GLUT 4
b-actin
2
NS
GLUT-4/b-actin levels
(fold increase over basal)
1
0
Ctr
20’
48h
EXE
C
GLUT4
### Chart
| Category | |
|---|---|GLUT-4 PM translocation
 (fold increase over basal)
Ctr
20’
48h
EXE
IR b
